# SCGNet: efficient sparsely connected group convolution network for wheat grains classification

**DOI:** 10.3389/fpls.2023.1304962

**Published:** 2023-12-22

**Authors:** Xuewei Sun, Yan Li, Guohou Li, Songlin Jin, Wenyi Zhao, Zheng Liang, Weidong Zhang

**Affiliations:** ^1^ School of Information Engineering, Henan Institute of Science and Technology, Xinxiang, China; ^2^ School of Artificial Intelligence, Beijing University of Posts and Telecommunications, Beijing, China; ^3^ School of Internet, Anhui University, Hefei, China

**Keywords:** wheat grains classification, feature multiplexing, sparsely connected, 3-D convolution, the number of parameters

## Abstract

**Introduction:**

Efficient and accurate varietal classification of wheat grains is crucial for maintaining varietal purity and reducing susceptibility to pests and diseases, thereby enhancing crop yield. Traditional manual and machine learning methods for wheat grain identification often suffer from inefficiencies and the use of large models. In this study, we propose a novel classification and recognition model called SCGNet, designed for rapid and efficient wheat grain classification.

**Methods:**

Specifically, our proposed model incorporates several modules that enhance information exchange and feature multiplexing between group convolutions. This mechanism enables the network to gather feature information from each subgroup of the previous layer, facilitating effective utilization of upper-layer features. Additionally, we introduce sparsity in channel connections between groups to further reduce computational complexity without compromising accuracy. Furthermore, we design a novel classification output layer based on 3-D convolution, replacing the traditional maximum pooling layer and fully connected layer in conventional convolutional neural networks (CNNs). This modification results in more efficient classification output generation.

**Results:**

We conduct extensive experiments using a curated wheat grain dataset, demonstrating the superior performance of our proposed method. Our approach achieves an impressive accuracy of 99.56%, precision of 99.59%, recall of 99.55%, and an *F*_1_-score of 99.57%.

**Discussion:**

Notably, our method also exhibits the lowest number of Floating-Point Operations (FLOPs) and the number of parameters, making it a highly efficient solution for wheat grains classification.

## 1 Introduction

Wheat, being one of the most extensively cultivated crops globally (Li et al., 2019; Zhou et al., 2021), holds vital genetic and morphological information within its seeds. The distinct characteristics and values exhibited by different wheat grain varieties underscore the importance of selecting high quality varieties. This selection is not only pivotal for augmenting wheat yields and enhancing quality but also crucial for safeguarding crops against pests and diseases (Mefleh et al., 2019; Saeed et al., 2022).

The purity of wheat grain varieties is of paramount importance to breeding specialists, wheat cultivators, and consumers at large (Hussain et al., 2022). Unfortunately, the integrity of seed markets faces challenges from unscrupulous traders who engage in deceptive practices. They market low-quality seed varieties as high-quality ones, posing a threat to consumers and disrupting the seed market. To counteract such issues, accurate classification techniques are imperative (Fanelli et al., 2023).

Historically, professionals relied on traditional methods for varietal identification of wheat grains. However, these methods are slow, labor-intensive, and susceptible to subjective biases. The inherent similarity in the characteristics of various wheat grains further complicates the identification process.

In recent years, the integration of computer vision techniques into wheat grain recognition has witnessed significant advancements (Li et al., 2019). Researchers have explored two primary types of approaches for feature extraction and classification: machine learning-based methods and deep learning-based methods.

Machine learning methods, while effective, require substantial agricultural knowledge, manual feature selection, and classifier design (Lu et al., 2022). This process demands significant human effort and may not match the recognition speed achieved by deep learning approaches.

Deep learning methods offer notable advantages, automating feature extraction and achieving superior classification accuracy. They exhibit strong generalization capabilities, streamlining model training and significantly enhancing recognition speed. However, challenges such as the need for extensive training data and the high number of parameters in deep learning models can impede deployment on resource-constrained devices. For instance, the computational intensity associated with these models can overwhelm devices with limited resources, leading to frequent crashes during usage. The sheer volume of computations required may exceed the processing capacity of these devices, compromising their stability and usability. Moreover, resource-constrained devices may lack the storage capacity necessary to accommodate the extensive parameters of these models, rendering deployment infeasible.

To address these challenges, we propose the Sparsely Connected Group Convolution Network (SCGNet) for efficient and accurate wheat grain classification. Our model is designed to offer a non-destructive, efficient, and rapid classification solution, aligning with the overarching goal of addressing the complexities associated with wheat grain identification and classification. We highlight the key contributions of this paper as follows:

- We introduce a novel approach known as “Group Mixing(GM)”, which involves splitting and rearranging group convolutions based on a strategic criterion. This innovative technique resolves issues related to information exchange among groups, enhances feature multiplexing, and simultaneously reduces the Floating-Point Operations (FLOPs) of the convolutional layers.
- We present a method for connected group convolutions, called “Sparsely Connected(SC)”, facilitating the cascading transfer of feature information between groups without compromising vital details. This advancement further decreases the computational demands of the convolutional layers.
- We incorporate 3-D convolution and revamp the convolutional classification layer within SCGNet. This novel approach replaces traditional layers like pooling and fully connected layers commonly found in conventional convolutional neural networks (CNNs). The result is a reduction in the overall number of model parameters, leading to a more streamlined architecture and faster recognition.

The structure of this paper is organized as follows: Section 2 provides a brief review and summary of various methods employed for wheat grain recognition, along with the challenges they address. In Section 3, we present comprehensive details regarding the proposed SCGNet architecture. Section 4 encompasses our creation of a wheat grains dataset, outlines our experimental procedures, and presents the experimental results. We also perform an in-depth analysis and comparison of classification outcomes generated by various network models using the wheat grain dataset. Finally, in Section 5, we summarize the primary contributions of this paper and engage in a discussion regarding potential directions for future research.

## 2 Related works

Currently, various identification methods have been gradually applied to wheat grain classification, and in the following, we provide an overview and summary of these studies and summarize the advantages and disadvantages of all methods in **Table 1**.

**Table 1:** Advantages and disadvantages of different methods.

Methods	Scholar	Advantages	Disadvantages
Machine learning	(Delwiche et al., 2013)	No extensive data sets are necessary for training, resulting in decreased computational resource requirements.	Scholars must possess a pertinent agricultural knowledge background and manually choose suitable features.
	(Güneş et al., 2014)		
	(Kurtulmuş et al., 2016)		
	(Sabanci et al., 2017)		
	(Ni et al., 2019)		
Deep learning	(Kozłowski et al., 2019)	It can automatically extract features, possesses a strong ability to generalize, and does not require the design of special classifiers.	A large amount of training data is required and the number of high-precision neural network model parameters is large.wedge background and manually choose suitable features.
	(Javanmardi et al., 2021)		
	(Yang et al., 2021a)		
	(Zhao et al., 2022)		
Deep Learning and Hyperspectral	(Weng et al., 2021)	The extracted feature information is richer and more resistant to interference.	High-quality equipment for collecting data and large datasets for training are required.
	(Shen et al., 2021)		
	(Yang et al., 2021b)		
	(Zhang et al., 2022a)		

### 2.1 Machine learning-based methods

Machine learning-based methods leverage digital image processing techniques to preprocess data acquired from collected images, followed by manual feature design, feature extraction, and ultimately, classification and recognition employing suitable classifiers like Support Vector Machine (SVM). For instance, Delwiche et al. (Delwiche et al., 2013) employed optical-grade reflectors to capture wheat grain images for assessing surface damage. They parameterized kernel morphology and texture features from both main and reflected views, employing parametric (Linear Discriminant Analysis, LDA) and non-parametric (k-Nearest Neighbors, KNN) classification models, respectively. This approach achieved a recognition accuracy of up to 94%. Güneş et al. (Güneş et al., 2014) proposed a method for recognizing wheat varieties using digital image processing techniques. Their system extracted image features using the Gray Level Covariance Matrix (GLCM) and Linear Binary Pattern (LBP) methods, classifying them with a k-nearest neighbor classifier. Kurtulmuş et al. (Kurtulmuş et al., 2016) introduced a recognition method combining machine vision and neural networks. They calculated features from different color components and constructed a feature database using chili pepper seeds as the study object. Sequential feature selection methods with various criterion functions were employed to select effective features, achieving variety classification of eight pepper seeds with a Multilayer Perceptron (MLP) accuracy of 84.94%. Sabanci et al. (Sabanci et al., 2017) extracted four shape features, three color features, and five texture features, inputting these features into an artificial neural network (ANN) constructed as a multilayer perceptron (MLP), resulting in improved classification results. Ni et al. (Ni et al., 2019)designed an automatic maize surface defect inspection system. Initially, they pre-processed touching kernels using a novel k-means clustering guided curvature method, enhancing the identification of broken kernels and system robustness. Subsequently, they integrated a deep convolutional network into the system for detecting maize surface defects, achieving an accuracy of 98.2%.

### 2.2 Deep learning-based methods

Deep learning-based methods typically involve the construction of specialized deep learning models for recognition and classification tasks (Huang et al., 2017; Zhang et al., 2022b; Zhang et al., 2023). In these approaches, the deep learning model takes the original image data as input, processes it at the pixel level, and automatically extracts contextual information and global features from the image by employing various combinations of convolution and pooling operations. Finally, the model produces classification and recognition results through specific functions. For instance, Kozłowski et al. (Kozłowski et al., 2019) conducted a comparison of nine different CNNs for wheat grain classification. They used reference performance indicators such as training time, inference speed, and accuracy rates and compared them with traditional machine learning methods. The results showed that traditional methods achieved a relatively low classification accuracy of around 75%, whereas CNN methods achieved an accuracy exceeding 93%. Javanmardi et al. (Javanmardi et al., 2021) proposed a method that utilizes CNNs as generalized feature extractors, combined with artificial neural networks, for feature extraction and classification. They tested this approach on 2250 test samples, achieving a correct classification rate of 98.1% with a total processing time of 26.8 seconds. Deep learning-based methods can achieve satisfactory results in terms of accuracy metrics, but it is worth noting that these high-precision CNNs are accompanied by a high number of parameters and FLOPs, and thus some scholars have focused on how to thin the models.

One noteworthy approach is MobileNet (Howard et al., 2017), which introduces depth-wise separable convolutions, which split standard convolutions into depth-wise convolutions and point-wise convolutions, this reduces the number of parameters and computations significantly. ShuffleNet (Zhang et al., 2018) employs group convolutions and channel shuffling to enhance the fusion of channel information while reducing computational cost, group convolutions split the input channels into separate groups, reducing the complexity of convolutions. MobileNetV2 (Sandler et al., 2018) utilizes the inverted residuals structure, which helps in maintaining a balance between computational efficiency and representational power, it uses linear bottlenecks and shortcut connections to improve information flow. Moreover, BiSeNetV2 (Yu et al., 2021) presented a branching network where the detail branch focused on underlying details using a larger spatial dimension, while the semantic branch captured advanced semantics with large convolutional kernels, these branches were then fused through an aggregation layer, enhancing the model’s capabilities. Inspired by these innovations, Yang et al. (Yang et al., 2021a) devised a branch network by modifying the VGG16 model. By removing the fully connected layer and adjusting the position of the Batch Normalization (BN) layer, they crafted a novel network capable of classifying peanut varieties. This tailored model exhibited remarkable accuracy improvements over the original design while maintaining a reduced parameter count. Zhao et al. (Zhao et al., 2022) employed YOLOv5 for detecting the quality of wheat grains and introduced a lightweight wheat grain detection network, WGNet, based on YOLOv5. WGNet utilized the FPN neck module and hybrid attention module to address performance degradation issues and reduced network parameters through network pruning. This approach significantly improved inference speed while maintaining high detection accuracy.

### 2.3 Methods based on hyperspectral imaging combined with deep learning

Neural networks have demonstrated outstanding performance, prompting some scholars to explore their combination with hyperspectral imaging techniques. For instance, Weng et al. (Weng et al., 2021) aimed to characterize the degree of Fusarium head blight (FHB) infestation on wheat grains. They extracted reflectance spectra from hyperspectral images of healthy and FHB-infected wheat grains with varying levels of infestation (light, moderate, heavy). Five effective wavelengths (EWs) were randomly selected from the spectra, and different combinations of EWs were used to generate reflectance images (RIs) with LeNet-5. Additionally, a residual attention convolutional neural network (RACNN) was constructed, increasing width and depth, and incorporating channel attention and residual modules to recognize varying degrees of FHB infection in wheat grains. Shen et al. (Shen et al., 2021) proposed a spectral imaging-based method for detecting impurities in wheat. They employed spectral imaging to study the spectral features of the target data and converted them into frequency domain spectra for terahertz pseudo-color imaging of wheat and its impurities. This was combined with a CNN to create a model called Wheat-V2, designed for identifying impurities in wheat images. Zhang et al. (Zhang et al., 2022a) utilized 2D convolution with an attentional mechanism to extract spatial and textural features, while 3-D convolution was used for spatial and inter-spectral information extraction in maize cultivar identification. This combination of 2-D and 3-D convolution showed good feature extraction complementarity. However, classification methods based on hyperspectral imaging often require a substantial amount of data and high-quality equipment for data acquisition. This instrument-dependent nature can be a burden for economically underdeveloped regions where the high cost of multispectral and hyperspectral cameras is prohibitive for widespread adoption. To address this, some scholars (Yang et al., 2021b) designed the Spectrogram Generative Adversarial Network (SPGAN) to expand the wheat grain dataset. SPGAN utilizes a generative adversarial network to generate synthetic datasets based on a small set of real datasets. These synthetic datasets serve as the foundation for the Progressive Neural Structure Search (PNAS) generative network structure, which classifies three types of wheat grains. The SPGAN-PNAS framework achieved an *F*_1_-score of 96.2%, outperforming traditional neural networks.

To summarize, machine learning-based methods do not require much data to have better performance although they need to extract features manually, while deep learning-based methods require a large amount of data to support them, at the same time, deep learning-based methods eliminate the need to manually design and extract features, which means that researchers do not need to have a richer background in agricultural knowledge. Hyperspectral-based methods combined with deep learning can extract richer feature information but require sophisticated data acquisition equipment.

## 3 Proposed SCGNET

**Figure 1:**
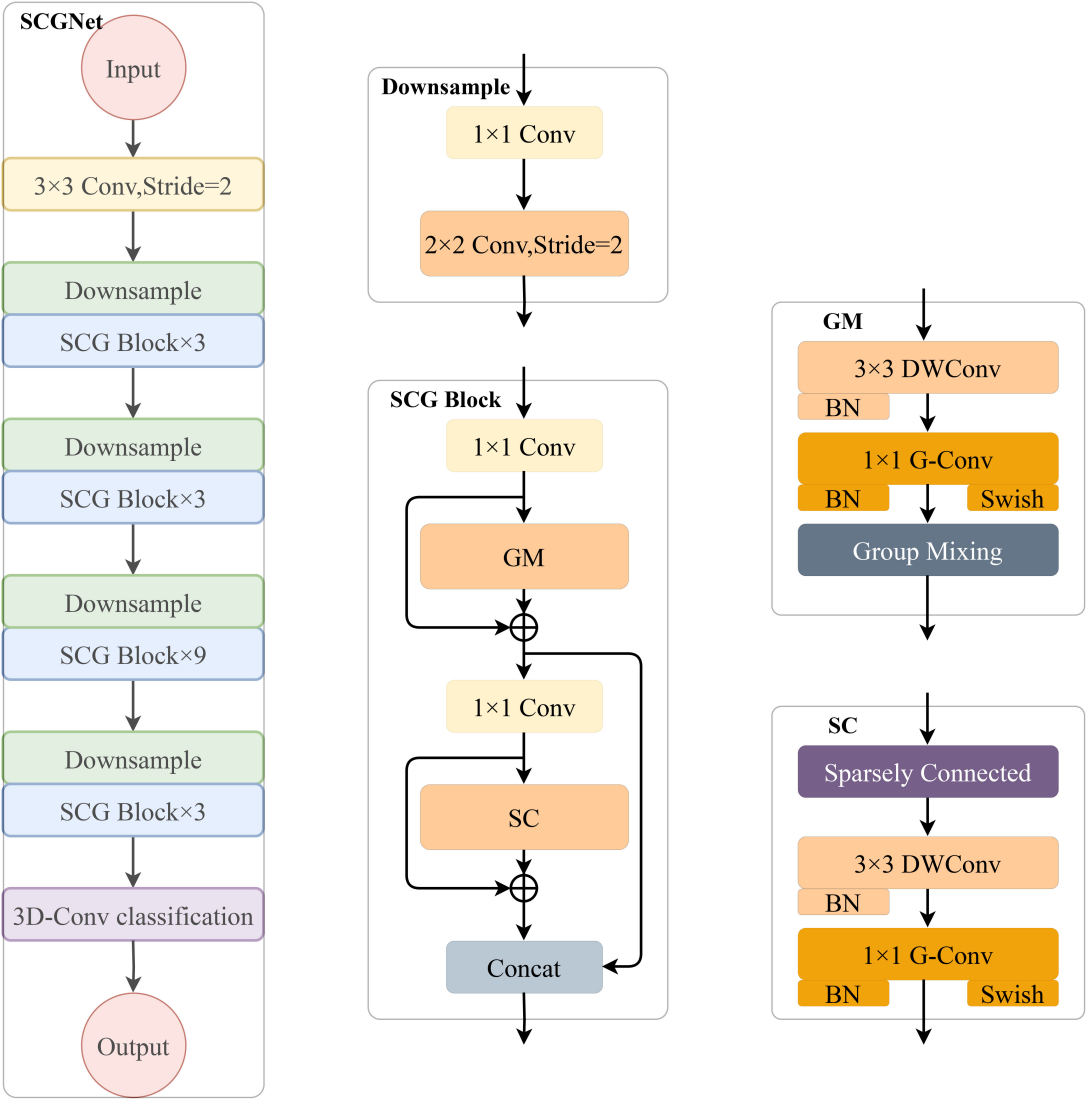
Given a 224×224×3 image of wheat grain, it passes through an initial convolutional layer to extract the coarse-grained features. Following that, we stack four successive SCG blocks to obtain the fine-grained features of the image and reduce the parameters. In addition, we add down sampling modules before each SCG block module to ensure image size consistency in the computation process. Finally, the output classification result is determined by a 3-D convolutional classification layer that we have constructed.

**Figure 1** provides an exhaustive overview of the proposed innovative SCGNet architecture. The left segment of the figure illustrates the overall structure of SCGNet, while the central part delves into the constituent sub-modules, namely the Downsample layer and SCG block. The right-hand portion zooms in further to unveil the sub-components of the SCG block. SCGNet is a comprehensive network composed of various elements, including a conventional convolutional layer, multiple repetitively stacked Downsample layers, SCG blocks, and a 3-D classification convolutional layer. The primary objective of these components is to capture and process the intricate features of the input image data.

The Conventional Convolutional Layer utilizes a 3×3 convolutional kernel with a stride of 1. Its role is to perform an initial extraction of the coarse-grained features inherent in the image data.

The Downsample Layer is crucial in reducing image size and controlling channel dimensionality. It consists of two convolutional layers: one using a 1×1 kernel with a stride of 1, and the other utilizing a 2×2 kernel with a stride of 2. This combination enables control of image size and channel simultaneously.

The SCG block represents the core of our architecture, consisting of a series of repetitively stacked down sampling layers and SCG block components(GM module and SC module). More details about the important sub-modules of the SCG block: the GM module and the SC module, will be elaborated upon in subsections 3.1.1 and 3.1.2. Additionally, it incorporates two 1×1 ordinary convolutional layers to regulate channel dimensions. The primary purpose of the SCG block is to extract fine grained features from the image and simultaneously reduce the overall parameter count of the entire SCGNet, thereby enhancing efficiency.

The 3-D Convolutional Classification Layer is specifically designed for classification tasks and employs 3-D convolutions to produce the final classification results.

In **Table 2**, we present a detailed breakdown of each module within SCGNet, offering a comprehensive reference for the configuration and specifications of our network’s components.

**Table 2:** Detailed specifications for each module within SCGNet.

Input	Layers	Kernel size	Stride	Channel	Repeat
224×224	Conv2d	3×3	2	3	1
112×112	Downsample	2×2	2	3	1
56×56	SCGNet-block	3×3	1	96	3
56×56	Downsample	2×2	2	96	1
28×28	SCGNet-block	3×3	1	192	3
28×28	Downsample	2×2	2	192	1
14×14	SCGNet-block	3×3	1	384	9
14×14	Downsample	2×2	2	384	1
7×7	SCGNet-block	3×3	1	768	3
7×7	3-D Conv Classification	3×3	1	768	1
Total Trainable Parameters: 1,078,091.					

### 3.1 SCG block

The SCG block serves as the main module of the entire network. Within the SCG block, we integrate the Group Mixing module and the Sparsely Connected module, along with two convolutional layers using 1×1 kernel sizes, to constitute the SCG block. These two 1×1 kernel convolutional layers play distinct roles: one for increasing and the other for decreasing the channel dimensionality. The GM module and SC module serve as the core components within the SCG block. Following the Depthwise Convolution (DW Conv), we introduce a BN layer to normalize the data, thus contributing to the potential acceleration of CNN training. The 1×1 Group convolution (G-Conv) primarily serves the purposes of parameter sharing and feature interaction among subgroups.

This aids in reducing the model’s parameter count while facilitating mutual feature learning among subgroups. After the G-Conv layer, we not only apply the BN layer but also opt for the Swish activation function over the traditional ReLU. The Swish activation function has a smoothness that enhances the forward propagation optimization, in addition, the function exhibits a high saturation threshold, which remains unsaturated even when the inputs converge to 0, thus facilitating the flow of gradients during the training process.

In various deep learning architectures, the concept of feature fusion is crucial. Such as ResNet (He et al., 2015) and FPN (Lin et al., 2017), these architectures often employ the element-wise Add operation for feature fusion.

The Add operation directly combines the matrix information from input features and output features without altering the image’s dimensionality. The number of channels remains the same, but the operation increases the amount of information along each dimension. However, in certain cases, such as ShuffleNet (Zhang et al., 2018), a Concat operation is used instead of Add during feature fusion.

The Concat operation, unlike Add, applies different weights to feature maps and then merges them based on the number of channels in the input matrix. This can increase the image’s dimensionality while preserving information along each dimension. The Concat operation aligns input features with the output feature map and leverages the semantic information from feature maps of different scales to achieve superior performance by expanding the number of channels. Therefore, to leverage semantic information from feature maps of different scales and increase dimensionality, we employ the Concat for feature fusion.

#### 3.1.1 Group mixing

Traditional CNNs primarily consist of convolutional layers, activation functions, pooling layers, and fully-connected layers (Krizhevsky et al., 2012). The trainable layers within CNNs typically comprise convolutional layers and fully-connected layers (Gao et al., 2018). Among them, the main role of the convolutional layer is to perform feature extraction on the input image. Each neuron in a convolutional layer is connected to multiple neurons in spatially proximate regions of the preceding layer. The convolution operation involves sequentially applying a convolution kernel to the input features through element-wise matrix multiplication and aggregating the results while incorporating biases.

The presence of a large number of convolution operations in convolutional layers leads to a substantial increase in the number of parameters and FLOPs. To illustrate this, let’s define the input feature map as *F* ∈ ℝ*^h^* ×*^w^* ×*^c^*, and the convolution kernel as *K* ∈ ℝ*^h^* ×*^w^* ×*^c^*, with *K_n_* representing the number of convolution kernels. A standard convolutional operation is performed between the feature map and *K_n_* convolutional kernels, with a default stride of 1. The number of parameters for this operation is calculated as Equation 1:


(1) $$\text{Parameters}=K_{h}\times K_{w}\times F_{c}\times K_{n}$$

At this point, the FLOPs are determined as Equation 2:


(2) $$\text{FLOPs}=K_{h}\times K_{w}\times F_{c}\times F_{h}\times F_{w}\times K_{n}$$

Here, *F_h_* and *F_w_* represent the height and width of the input feature map, respectively. *K_h_* and *K_w_* denote the height and width of the convolution kernel, *F_c_* is the number of channels in the feature map, and *K_c_* is the number of channels in the convolution kernel. in which case, *K_c_* = *F_c_*. To address this computational complexity, MobileNet (Howard et al., 2017) introduced depthwise separable convolution. This technique divides the convolution operation into two steps: depthwise convolution and pointwise convolution. In the depthwise convolution, a single convolution kernel is applied independently to each channel in the depth direction of the feature map. The outputs are then concatenated to generate the same number of output channels, followed by pointwise convolution using a 1×1 unit convolution kernel. With this decomposition, the number of convolutional parameters as Equation 3:


(3) $$\text{Parameters}=K_{h}\times K_{w}\times F_{c}+F_{c}\times K_{n}$$

And the FLOPs as Equation 4:


(4) $$\text{FLOPs}=K_{h}\times K_{w}\times F_{h}\times F_{w}\times F_{c}+F_{c}\times F_{h}\times F_{w}\times K_{n}$$

Clearly, there is a significant reduction in both parameters and FLOPs after applying depthwise convolution and pointwise convolution. However, constrained by the computational power of GPUs, there is a need to further reduce parameters and FLOPs. To address this, AlexNet (Krizhevsky et al., 2012) introduced group convolution. This technique involves grouping different feature maps of the input layer and applying different convolution kernels to each group. Group convolution has been successfully employed in various networks, including Xception (Chollet, 2017), MobileNet (Howard et al., 2017), ResNeXt (Xie et al., 2017), and others, demonstrating excellent performance.

Group convolution is a technique used in CNNs to reduce the computational cost of convolutional layers. It divides the input feature map into mutually exclusive groups based on channels, where each group operates with a 1×1 convolution kernel. This division results in each group having a subset of the input channels, with a proportionate reduction in the number of parameters. The number of parameters and FLOPs are reduced to 1/G of the original values, where G is the number of groups.

However, group convolution also brings an Issue of Independence. While group convolution significantly reduces computational requirements, it has a drawback. The feature information in each subgroup is relatively independent, and there is limited interaction between the groups. This can lead to a lack of effective information exchange between channels.

To address the issue of independence and enhance information exchange between the groups, we propose a “Group Mixing” approach. First, group convolution is divided into G primary groups (*G_i_*). Each primary group contains a subset of the channel’s feature information. Then, each primary group (*G_i_*) is further divided into j subgroups ( $G_{i}^{j}$ ), where *j* ranges from 1 to *i*. This secondary division allows for a more fine-grained separation of channel information within each primary group. Finally, the critical step in Group Mixing involves taking one subgroup ( $G_{i}^{j}$ ) from each primary group (*G_i_*) and combining them in an ordered manner. These subgroups are concatenated to create new subgroups ( ${\hat{G}}_{i}$ ) in a way that disrupts and recombines the feature information.

By using the Group Mixing method, feature information from each primary group is mixed in an orderly manner to generate new groups ( ${\hat{G}}_{i}$ ). For example, *G*_1_ contains information from all $G_{i}^{j}$ . This process enhances the interaction and information exchange between different groupings and channels. For the new *G*_1_, it comprises each $G_{1}^{j}$ component, and each new ${\hat{G}}_{i}$ mixed group is defined as Equation 5:


(5) $${\hat{G}}_{i}=\sum_{j=1}^{j}G_{i}^{j}$$

**Figure 2:**
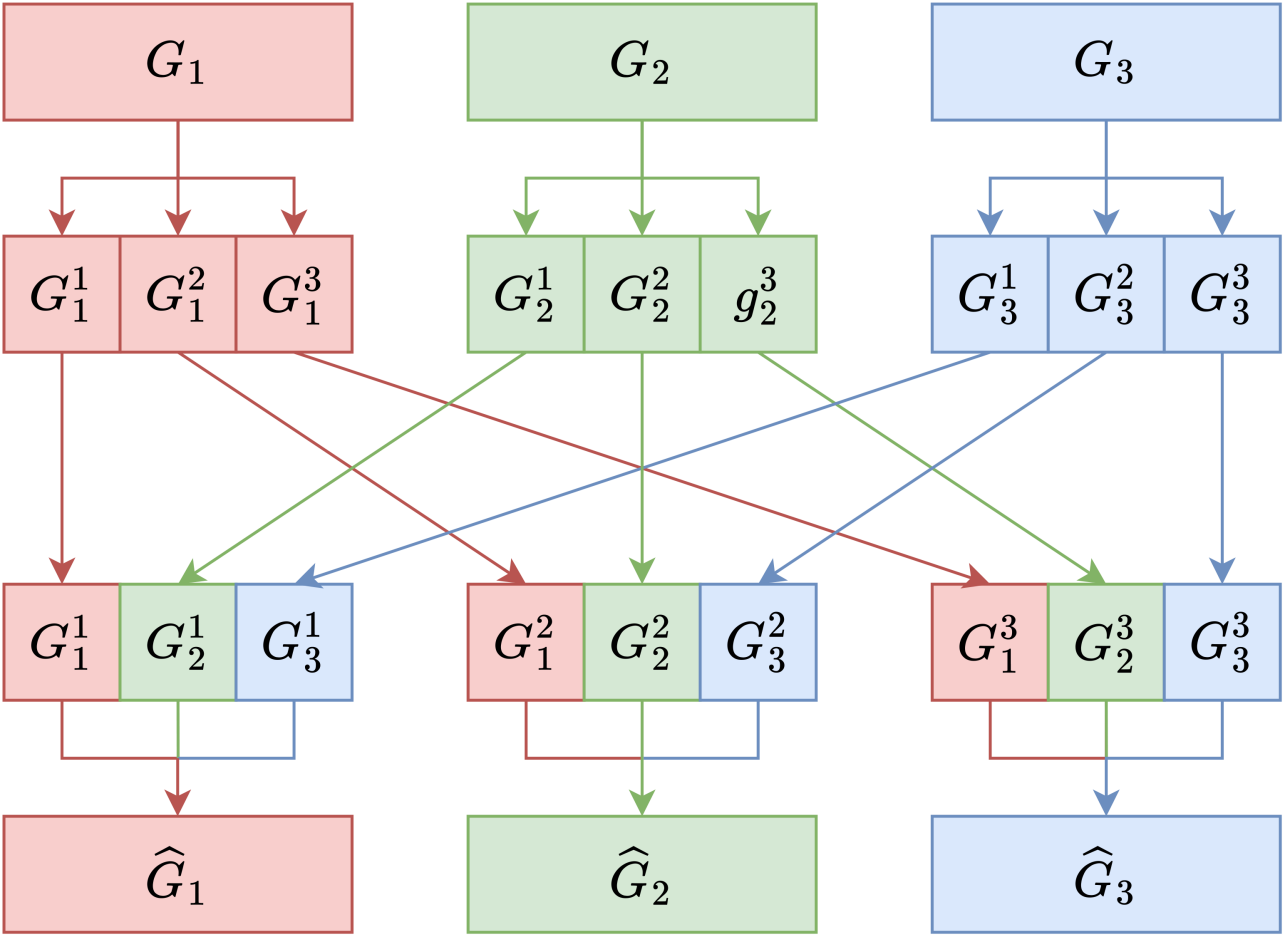
Schematic diagram of Group Mixing transformation.

Group Mixing is a strategy to balance computational efficiency (achieved through group convolution) with the need for information exchange and interaction between feature channels, particularly in the context of group convolution. This helps in maintaining the representational power of the network while reducing computational complexity. It disrupts and recombines feature information in an ordered manner, allowing for more effective interaction between subgroups, thus addressing the issue of independence observed in group convolution. **Figure 2** provides a visual representation of our proposed method.

#### 3.1.2 Sparsely connected

**Figure 3:**
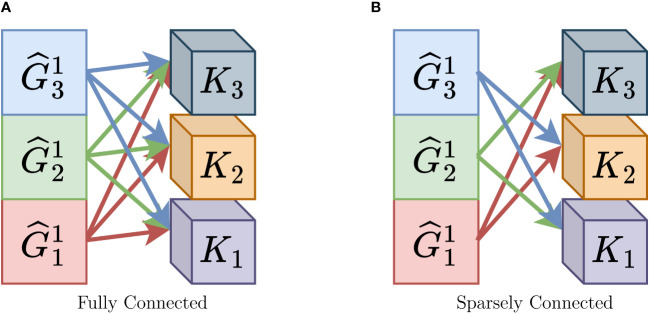
Difference between Fully Connected and Sparsely Connected. **(A)** Fully Connected, **(B)** Sparsely Connected.

Traditionally, in CNNs, the output of group convolutions is connected to the subsequent layer in a manner that resembles a fully connected layer, as depicted in **Figure 3A**. This design choice is made to ensure that most of the feature information is preserved since there is typically no information exchange among individual subgroups. As a result, dense connections are used to pass feature information to the next layer. However, with the introduction of “Group Mixing” as discussed in Section 3.1.1, the problem of information exchange between group convolutions has already been addressed to a significant extent, making the dense connections unnecessary. In the essence of convolutional operations, where a feature map is convolved with a kernel, the operation spans both height and width dimensions, constituting a spatial convolution. The 1×1 convolution operation, often utilized in CNNs, is equivalent to a fully connected operation. Building on this understanding, Sparsely Connected offers an alternative perspective on the convolution operation, specifically in the channel dimension.

Building on our previous discussion, when the input feature map *F* ∈ ℝ*^h^* ^×w×^ *^c^* is convolved with the convolution kernel *K* ∈ ℝ*^h^* ^×w×^ *^c^*, this convolution is equivalent to a fully connected operation in both the spatial dimensions and the channel dimension. With this understanding, we introduce “ Sparsely Connected,” a method primarily aimed at reducing the number of fully connected operations. When the input feature map *F* ∈ ℝ*^h^* ^×w×^ *^c^* is convolved with the convolution kernel *K* ∈ ℝ*^h^* ^×w×^ *^c^* in the channel dimension, we no longer perform a fully connected operation. Instead, we adopt a sparsely connected approach in the channel dimension by employing a certain stride. Simply put, the feature map is convolved with only a part of the convolution kernels. For instance, with stride=3, 2 convolution kernels after one convolution operation are discarded. Regarding the choice of stride, we conducted a series of comparative experiments in the ablation study in Section 4.5, as shown in **Table 3**, verifying that the best performance is achieved when stride = 3. Also, as the stride gets larger, the model exhibits worse performance.

**Table 3:** Results of the sparsely connected method when different strides are taken for ablation studies of SC modules.

Methods	Accuracy↑	FLOPs↓	Parameters↓
SC, stride=2	99.56%	34.97 M	1.06 M
SC, stride=3	99.56%	34.43 M	1.03 M
SC, stride=5	97.08%	33.91 M	1.01 M
SC, stride=7	94.27%	30.18 M	0.94 M
SC, stride=9	86.25%	28.67 M	0.89 M

(Optimal: red Suboptimal: blue).

↑ indicates that the larger the value of the item, the better, and ↓ indicates that the smaller the value of the item, the better.

By implementing Sparsely Connected, we eliminate the necessity for *F_c_* to be multiplied by *K_n_*, thereby significantly reducing the computational burden associated with convolution operations while preserving the essential information required for subsequent processing.

Based on the previous description, we can integrate “Sparsely Connected” with “Group Mixing”.

In the after of Group Mixing, we obtain *K_n_* feature maps following group convolution. Subsequently, we partition these *K_n_* feature maps into *g* groups and employ *g* independent convolution kernels *K* ∈ ℝ*^h^* ^×w×^ *^c^*. The step size between feature maps and convolution kernels for each convolution operation is set to *g*, and *K_c_* ≤ *F_c_*. By performing convolution on the entire input *K_n_/g* times, the number of feature maps is reduced to *K_n_/g*. At this juncture, the parameter count for convolutional computation is calculated as Equation 6:


(6) $$\text{Parameters}=\frac{F_{c}}{g}\times \frac{K_{n}}{g}\times g+K_{c}\times g$$

The FLOPs are determined as Equation 7:


(7) $$\text{FLOPs}=\frac{F_{c}}{g}\times \frac{K_{n}}{g}\times F_{h}\times F_{w}\times g+K_{c}\times \frac{K_{n}}{g}\times F_{h}\times F_{w}\times g$$

Clearly, by incorporating the “Sparsely Connected” approach, we further reduce the number of parameters in the CNN, enabling it to operate more efficiently. Moreover, this approach facilitates the seamless transfer of feature information from one layer to the next without compromising the effectiveness of the feature information. **Figure 3B** nicely illustrates our proposed sparsely connected approach.

### 3.2 3-D convolutional classification layer

The classical structure of a CNN typically includes convolutional layers, pooling layers, and fully connected layers. Traditionally, these networks used average pooling and multiple fully connected layers. Earlier networks like AlexNet (Krizhevsky et al., 2012), VGGNet (Simonyan and Zisserman, 2015), and GoogLeNet (Szegedy et al., 2015), for instance, featured three consecutive fully connected layers. However, these fully connected layers contained a large number of parameters due to their fully connected nature. In fact, in the case of AlexNet (Krizhevsky et al., 2012) on the ImageNet dataset, the three fully connected layers accounted for approximately 96% of the total number of parameters in the entire network, which is nearly the sum of all parameters in the network. Importantly, experimental results demonstrated that this design did not substantially compromise the classification performance of the CNN.

Many studies have revealed that the weight matrix of these fully connected categorization layers is often very sparse, suggesting that only a few features are essential for category prediction. The problem of excessively large fully connected layers has garnered attention from researchers. Consequently, in recent works, (Tan and Le, 2020; Dai et al., 2021; Liu et al., 2022), the last two fully connected layers in the network structure were replaced with a global average pooling layer followed by a single fully connected layer. This modification significantly reduced the total number of parameters in the CNN. For example, in the case of the lightweight network MobileNet (Howard et al., 2017) on the ImageNet dataset, the fully connected layer accounted for approximately 24% of the total network parameters.

In the initial design of SCGNet, we also adopted this approach: replacing the last two layers with a single global pooling layer and a single fully connected layer. However, during the design process, we discovered that even with only one fully connected layer, it still accounted for a significant portion of the CNN’s parameters. This means that the number of parameters in the fully connected layer is still a large percentage of the overall CNNs. To address this, we propose a novel classification layer based on 3-D convolutional operations. This new layer completely replaces the traditional global pooling and fully connected layers, resulting in a reduction in the number of parameters and FLOPs in the network.

In a typical CNN, several convolutional layers are employed to progressively extract features essential for image recognition and classification through convolution operations. These convolutional layers output larger-sized feature maps. Subsequently, these feature maps are passed through a Global Average Pooling layer, which serves the purpose of further downsizing these large feature maps. After traversing multiple pooling layers, these feature maps are then used as inputs for the fully connected layer. The role of the fully connected layer is to connect each node to all nodes in the previous layer and ultimately output a one-dimensional vector. The classification result is obtained by applying the softmax classification function.

Based on our prior description of global pooling and fully connected layers, we can simplify the process when an input feature map *F* ∈ ℝ*^h^* ^×w×^ *^c^* passes through the global pooling layer as follows:

Firstly, we can streamline this step by employing *F_c_* convolution kernels, each with dimensions *F_h_* ×*F_w_* ×1. This implies that *F_h_* and *F_w_* remain consistent with *K_h_* and *K_w_*, respectively, while maintaining uniform weights set at 1*/F_h_* ×*F_w_*.

Secondly, to facilitate the seamless connection between the output of the pooling layer and the input of the fully-connected layer, we approximate the fully-connected layer by substituting it with a convolutional layer. The size of the convolutional kernel in this context becomes 1×1×*K_c_*.

**Figure 4:**
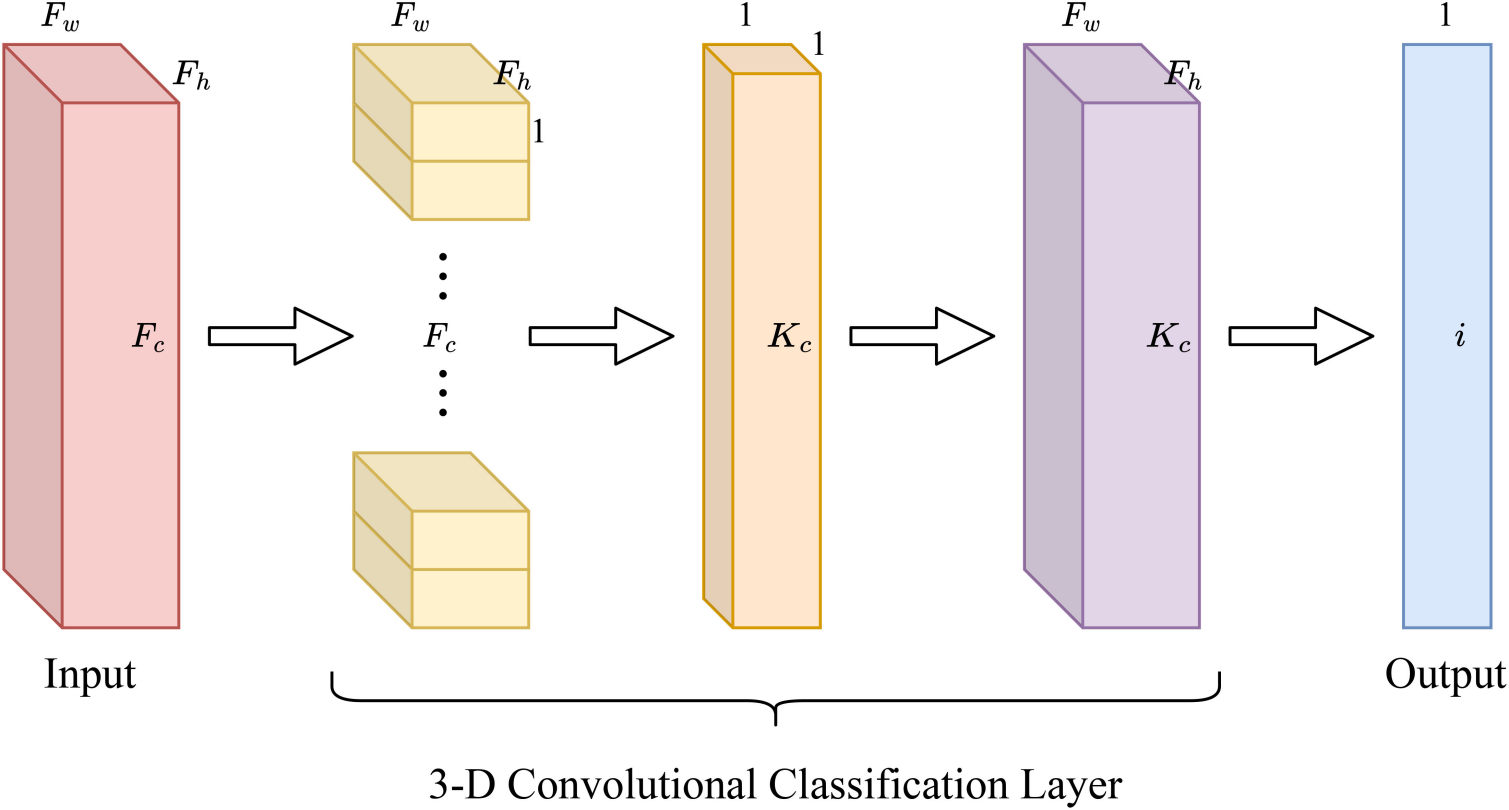
3-D convolutional classification layer.

Subsequently, we amalgamate these two convolutional operations, yielding a 3-D convolution operation with a convolutional kernel size of *F_h_* × *F_w_* × *K_c_*. This 3-D convolution layer is predominantly utilized in constructing the entire classification layer. This approach simplifies both the pooling layer and the fully connected layer into a single 3-D convolutional layer, as eloquently depicted in **Figure 4**.

When this integrated layer performs the classification task, assuming the number of categories to be classified is denoted as *i*, we adjust *K_c_* to be *F_c_* − *i* +1. In other words, the prediction for each category necessitates only *F_c_* − *i* +1 input feature maps. This disambiguation strategy optimally conserves computational resources within the CHANNEL dimension, thereby reducing parameters and computational complexity while preserving the essential category connections for accurate prediction and ensuring efficient categorization output.

Our proposed method combines global pooling and fully connected layers into a unified 3-D convolutional layer, resulting in significant computational efficiency gains without compromising classification accuracy. By implementing this approach, we significantly reduce computational resources in the CHANNEL dimension. This reduction aids in lowering the number of parameters and computations while preserving the connectivity necessary for category prediction and maintaining efficient categorization output.

## 4 Experimental results and analysis

In this chapter, we begin by introducing the wheat grains dataset that we have utilized, as well as detailing the preprocessing procedures it underwent. Subsequently, we delve into an exploration of the impact of specific parameter settings on the classification capabilities of SCGNet. Following that, we conduct a comprehensive comparison with a series of CNNs commonly employed for image classification. Our objective is to evaluate and highlight the advantages of our proposed SCGNet, with a focus on key metrics such as accuracy, parameters, FLOPs, and other relevant factors.

### 4.1 Dataset description

The dataset samples we utilized were sourced from the experimental field of the School of Life Science and Technology at Henan Institute of Science and Technology. These samples were generously provided by our colleagues at the School of Life Science and Technology. Following a careful evaluation, we specifically selected the following wheat varieties for inclusion in our dataset: “Bainong 419,” “Bainong 207,” “Bainong 307,” “Luomai 28,” “Xinmai 26,” “Hengshui 6632,” “Nongda 3416-18,” and “Neile 288.” These varieties represent commonly cultivated wheat types in China and serve as a comprehensive representation of wheat diversity.

**Figure 5:**
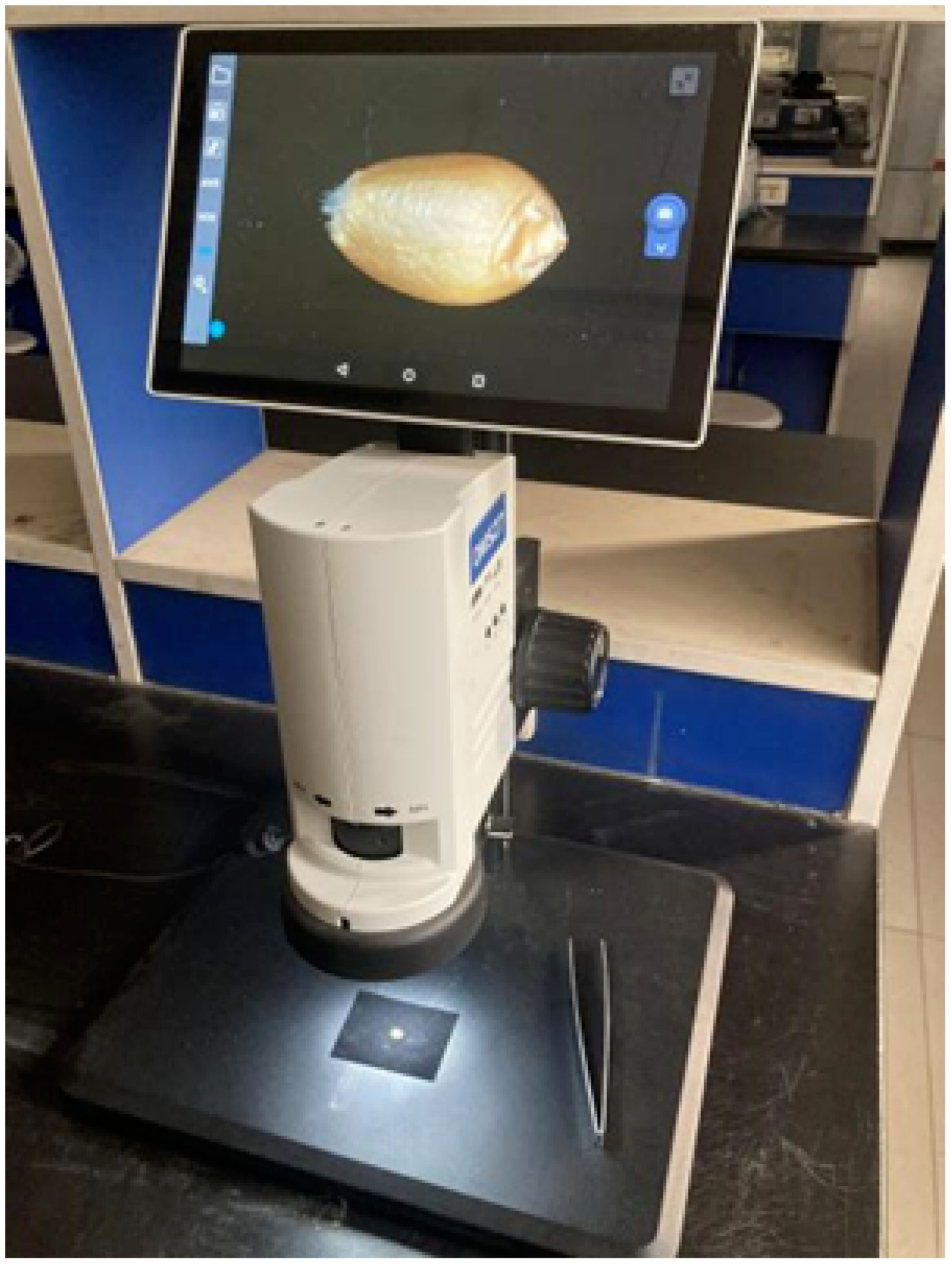
Image acquisition device: stereo microscope.

To capture high-quality images of these wheat samples, we employed a stereo microscope, as depicted in **Figure 5**.

To minimize any potential external environmental interference, we utilized black light-absorbing flannel as the background for capturing wheat grains images. The image collection process was conducted under natural indoor lighting conditions. For each wheat grain, we captured three images from different angles. It’s important to note that when assembling the dataset, wheat grains from the same variety but with varying angles were categorized together.

To ensure the quality and consistency of our dataset, a meticulous data collection process was employed. Initially, all wheat grains were subjected to a drying procedure in a well-ventilated indoor environment. Subsequently, a total of 8,000 seeds, with 1,000 grains selected from each wheat variety, were carefully handpicked. We chose three specific shooting angles for image capture: Ventral groove downward, Ventral groove toward the front, and Ventral groove upward. These images were saved in PNG format with a pixel resolution of 2688×1520. This comprehensive approach to data collection ensured the richness and completeness of our dataset, contributing to the robustness of our study. Consequently, we amassed a total of 24,000 images. The correspondence between each wheat variety and its corresponding number is detailed in **Table 4**.

**Table 4:** Correspondence between number, quantities and species name of wheat grains of different varieties in the dataset.

Number	Species name	Quantities
1	Bainong-207	3000
2	Bainong-419	3000
3	Hengshui-6632	3000
4	Luomai-28	3000
5	Neile-288	3000
6	Nongda-3416-18	3000
7	Xinmai-26	3000
8	Xunong-14084	3000

### 4.2 Data pre-processing

After the initial dataset collection, we diligently undertook a comprehensive dataset preprocessing pipeline. Our approach encompassed several crucial steps which are briefly described below (Zhuang et al., 2022).

**Figure 6:**
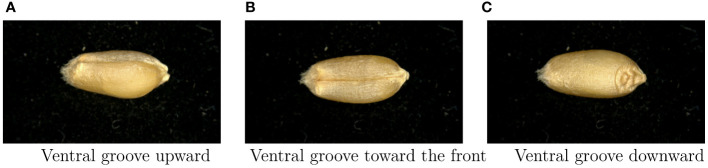
The presentation of the same sample in the dataset with different shooting angles, the image has high clarity and the interference of the background on the subject content is obvious. **(A)** Ventral groove upward, **(B)** Ventral groove toward the front, **(C)** Ventral groove downward.

**Figure 7:**
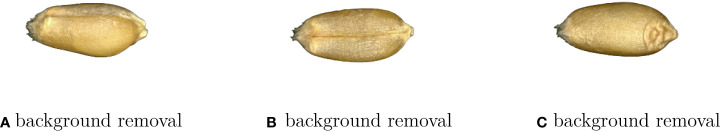
Using the advanced keying algorithm (Sengupta et al., 2020) (Lin et al., 2021) results. **(A–C)** correspond to **(A–C)** in **Figure 6**, respectively.

Background Removal: In **Figure 6**, noticeable artifacts such as small white dots and lines were discernible in the original images. These imperfections arose from the inherent characteristics of the stereo microscope, capturing minute particles like lint and dust during the imaging process. To mitigate the influence of these extraneous elements on the subject matter, we employed sophisticated keying algorithms known as Background Matting and Background Matting V2 (Sengupta et al., 2020; Lin et al., 2021) to effectuate background removal across our dataset. The image with the background removed is shown in **Figure 7**.

Image Resizing: The original images, as captured by the stereo microscope, featured dimensions of 2688×1520 pixels. Following the background removal in the previous step, we uniformly resized the images to 800×800 pixels, specifically focusing on isolating wheat seed grains. Subsequently, we further scaled down the image dimensions to 224×224 pixels, maintaining proportional scaling.

Standardization: To ensure consistency and facilitate convergence during training, we are based on experience standardized each image by setting the mean and standard deviation to (0.485, 0.456, 0.406) and (0.229, 0.224, 0.225), respectively. This standardization process was pivotal in optimizing the numerical properties of the images.

Dataset Split: For proper model evaluation, we randomly partitioned the dataset into the training set, validation set, and testing set with 7:1:2 ratio. This division allowed us to validate the model’s performance on unseen data, adhering to best practices in experimental design.

Transfer Learning: During the training phase, we employed transfer learning techniques by loading weight files pre-trained on the ImageNet dataset into our training model and the comparative models used in our experiments. This practice leveraged knowledge acquired from a large-scale dataset to enhance the performance of our models on the specific task at hand.

### 4.3 Evaluation criteria

In our evaluation of the network models, we employ several key metrics, including accuracy, precision, recall, and the *F*_1_-score, to assess the recognition performance of each model. The mathematical expressions for these metrics are provided in Equations 8–11.


(8) $$\text{Accuracy}=\frac{TP+TN}{TP+TN+FP+FN}$$

(9) $$\text{Precision}=\frac{TN}{FP+TN}$$

(10) $$\text{Recall}=\frac{TP}{TP+FN}$$

(11) $$F_{1}=\frac{2PR}{P+R}=\frac{2TP}{2TP+FP+FN}$$

**Figure 8:**
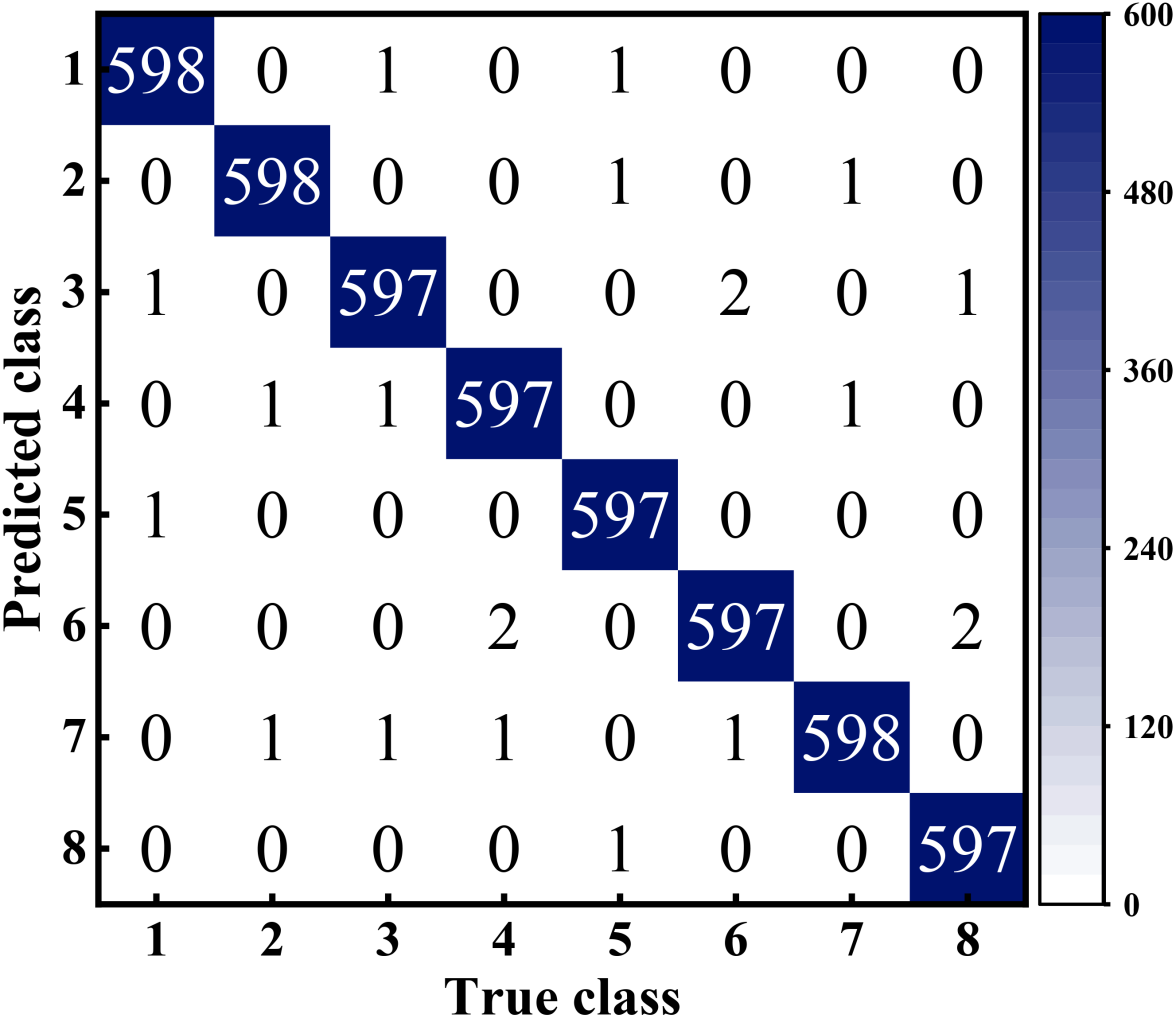
Confusion matrix of SCGNet for eight wheat grains classification results, Among them, the correspondence between number and type is detailed in **Table 4**.

Here, let’s clarify the definitions of the variables used in these equations. True Positive (TP) represents the instances that truly belong to a category and are correctly recognized by the classifier, while False Negative (FN) represents instances that belong to a category but are incorrectly categorized. On the other hand, False Positive (FP) signifies instances that do not belong to a category but are incorrectly recognized as belonging to that category, and True Negative (TN) corresponds to instances that do not belong to a category in reality and are correctly recognized as such. In addition, we show the confusion matrix (Srinivasu et al., 2022)of the experimental results in **Figure 8**.

### 4.4 Comparison experiment

Throughout our experiments, when assessing the performance of different networks, we consider not only recognition accuracy but also other critical metrics, such as the number of parameters, average recognition time, and FLOPs. These metrics hold particular importance in our work, as our primary focus is on reducing these values to enhance the feasibility of deploying these models on mobile devices, thus improving their speed and efficiency in mobile applications. To quantify these metrics, we utilized the open-source project torchstat, which allowed us to calculate parameters, FLOPs, and other relevant statistics for each network.

To assess the efficacy of our proposed SCGNet, we present a comprehensive analysis of nine deep learning models for image classification to assess their effectiveness and suitability for various practical applications. We employ a consistent training, validation and testing dataset to ensure a fair and robust comparison, focusing on evaluating key performance metrics such as accuracy, precision, recall, *F*_1_-score, FLOPs, the number of parameters, and average recognition speed.

Our analysis covers a spectrum of network architectures, including traditional models with classical design principles models such as ResNet50, EfficientNet and RegNetX, lightweight models optimized for resource-constrained environments such as MobileNetV3, and ShuffleNetV2, network models with a transformer structure such as Vision Transformer and Swin Transformer, as well as deep models aimed at achieving SOTA accuracy such as RepLKNet and MAGE.

**Figure 9:**
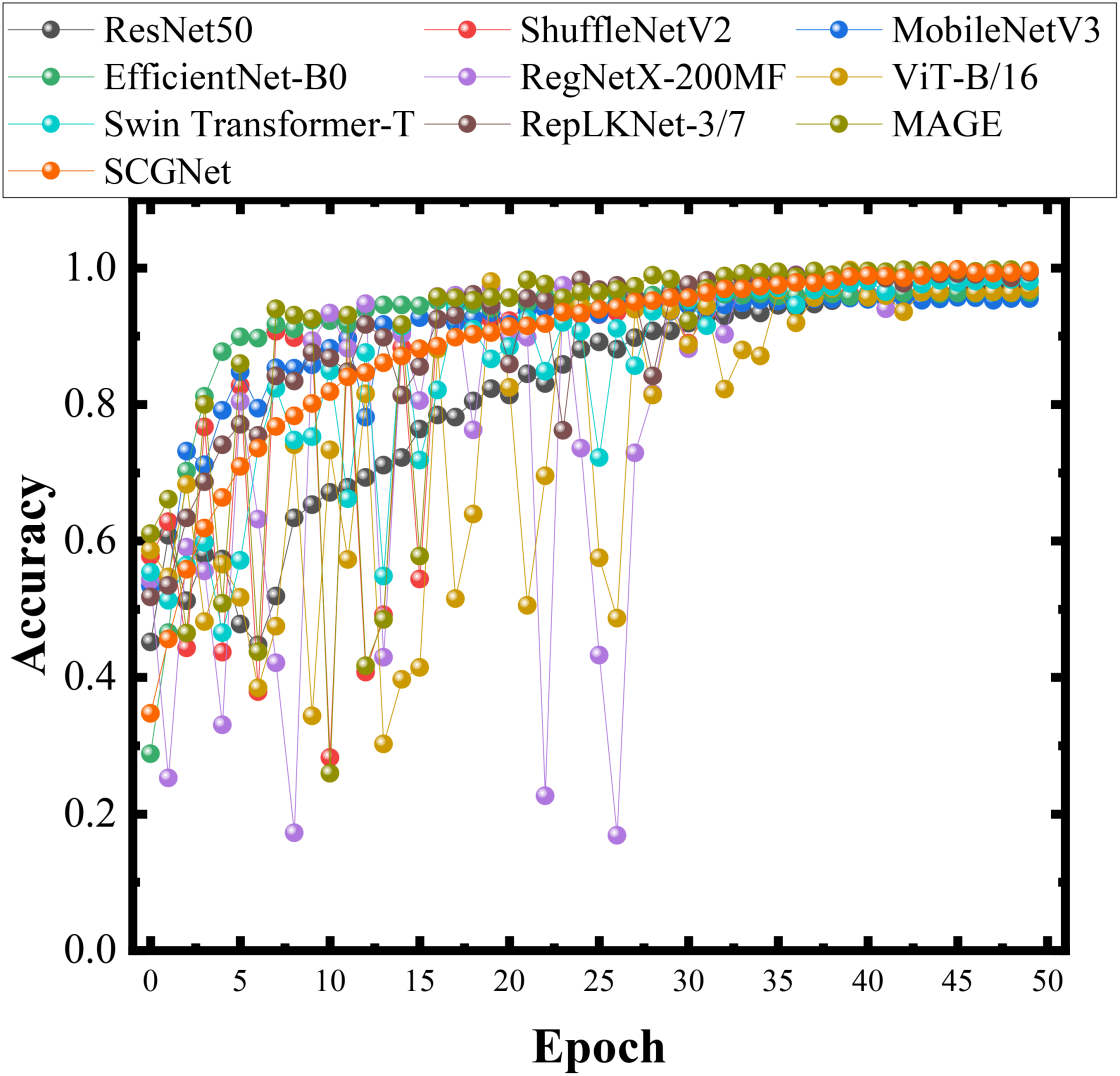
Histograms of different methods in the same data set during training.

**Figure 10:**
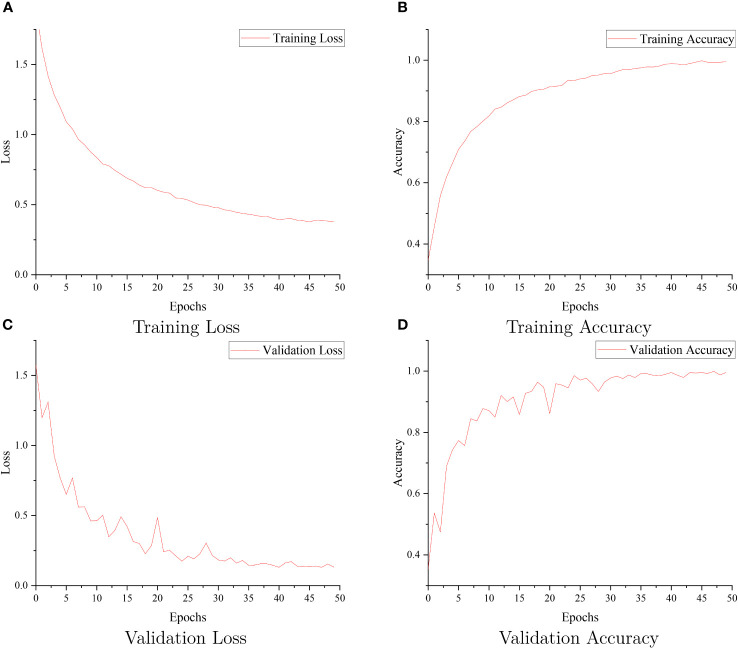
The SCGNet training loss **(A)**, training accuracy **(B)**, validation loss **(C)**, and validation accuracy **(D)**.

**Figure 9** visually presents the accuracy results obtained during training for each of these network models, providing an intuitive overview of their performance. Additionally, **Figure 10** reports the training loss, validation loss, training accuracy, and validation accuracy for SCGNet. **Tables 5**, **6** present a comprehensive summary of the results from the comparative experiments in the testset, encompassing various evaluation metrics.

**Table 5:** Differences between SCGNet and other comparison methods in terms of Accuracy, Precision, Recall and *F*_1_-Socre under the same testset.

Methods	Accuracy ↑	Precision ↑	Recall ↑	*F*_1_-Socre ↑
ResNet50 (He et al., 2015)	92.38%	92.58%	92.12%	92.35%
ShuffleNetV2 (Ma et al., 2018)	96.40%	96.21%	96.41%	96.31%
MobileNetV3 (Howard et al., 2019)	95.63%	95.31%	95.79%	95.55%
EfficientNet-B0 (Tan and Le, 2020)	97.77%	97.58%	98.02%	97.80%
RegNetX-200MF (Radosavovic et al., 2020)	97.71%	98.13%	97.51%	97.82%
ViT-B/16 (Dosovitskiy et al, 2020)	99.48%	99.45%	99.57%	99.51%
Swin Transformer-T (Liu et al., 2021)	99.50%	99.48%	99.56%	99.52%
RepLKNet-3/7 (Ding et al., 2022)	99.54%	99.43%	99.67%	99.55%
MAGE (Li et al., 2023)	99.58%	99.50%	99.67%	99.58%
SCGNet	99.56%	99.59%	99.55%	99.57%

(Optimal: red Suboptimal: blue).

↑ means that the larger the value of the item, the better.

**Table 6:** Differences between SCGNet and other comparison methods in terms of FLOPs, Parameters and Average recognition speed.

Methods	FLOPs↓	Parameters↓	Recognition speed↓
ResNet50 (He et al., 2015)	4 12 G	24.37 M	113 ms
ShuffleNetV2 (Ma et al., 2018)	43.65 M	1.30 M	63 ms
MobileNetV3 (Howard et al., 2019)	59.81 M	2.43 M	70 ms
EfficientNet-B0 (Tan and Le, 2020)	399.3 M	5.04 M	97 ms
RegNetX-200MF (Radosavovic et al., 2020)	203.75 M	2.56 M	89 ms
ViT-B/16 (Dosovitskiy et al., 2020)	6.15 G	38.62 M	127 ms
Swin Transformer-T (Liu et al., 2021)	8.33 G	49.42 M	159 ms
RepLKNet-3/7 (Ding et al., 2022)	12.9 G	76.57 M	213 ms
MAGE (Li et al., 2023)	20.66 G	179.24 M	164 ms
SCGNet	34.43 M	1.03 M	59 ms

(Optimal: red Suboptimal: blue).

↓ indicates that the smaller the value of the item, the better.

For comprehensive reference, we have meticulously documented all parameter settings utilized in the training of SCGNet. We use the validation set to evaluate the performance of the model with different parameter settings and finalize all the hyperparameters. These settings are presented in **Table 7**, allowing for a clear understanding of the experimental setup and facilitating reproducibility. In the comparative experiments involving different networks, since there are different versions of baseline and various improvements, we adhered to officially recommended parameter settings for these models to ensure consistency and fairness in our evaluations.

**Table 7:** SCGNet hyperparameter settings.

hyperparameter	Value
Optimizer	AdamW
Initial learning rate	4e-3
Weight decay	0.005
Optimizer decay	*β*_1_ = 0.9, *β*_1_ = 0.999
Batch size	64
Training epochs	50
Learning rate schedule	Cosine decay
Label smooth	0.1

For Traditional Network Models, while foundational in the field, displayed suboptimal performance compared to more recent innovations. ResNet50, with its increased depth and residual connections, improves recognition accuracy but still falls short due to its relatively shallow architecture. If the depth of the network is increased without restriction, although the model is able to achieve better performance, the number of parameters of the model will also increase dramatically. EfficientNet-B0 incorporated Neural Architecture Search (NAS) principles to amalgamate depth, width, and channel scaling, achieving formidable recognition capabilities but at the expense of increased parameter complexity. RegNetX-200MF refined the NAS approach, achieving substantial parameter reduction while maintaining accuracy, albeit with a slight deficit compared to SCGNet.

For Lightweight Network Models, including MobileNetV3, and ShuffleNetV2, demonstrated a harmonious balance between accuracy and computational efficiency. MobileNetV3, building upon its predecessor, introduced NetAdapt and various NetPruningVersions (NPVs) alongside an algorithm to optimize convolutional kernels and channels, further enhancing its performance. ShuffleNetV2 adopts a split-and-concatenate strategy to reduce overall computational demands.

For the Transfomer Network Models, namely Vision Transformer and Swin Transformer, their performance is already very close to that of SCGNet, and, Swin Transformer is capable of suboptimal performance in the precision rate metric. However, it should not be overlooked that they possess a huge number of parameters.

For the SOTA Network Models, include RepLKNet and MAGE. MAGE exhibits the highest accuracy, recall and *F*_1_-Socre, while RepLKNet displays the highest recall. They both exhibit exceptional precision performance. However, they place excessive emphasis on metrics such as accuracy, neglecting the balance between speed and precision, average recognition speed is relatively slow. Moreover, they have a large number of parameters, FLOPs, with RepLKNet and MAGE’s FLOPs being 384 and 614 times higher than that of SCGNet, respectively.

For SCGNet, the proposed SCGNet exhibits commendable performance in resource utilization metrics such as FLOPs, average recognition speed and the number of parameters, surpassing alternative models in these aspects. Despite its sub-optimal performance in accuracy and *F*_1_-Socre compared to the MAGE model, the marginal 0.02% difference in accuracy is deemed negligible. We maintain that sacrificing such a small improvement in accuracy for the reduction in FLOPs, average recognition speed and parameter count makes SCGNet highly cost-effective. This is especially favorable for the model’s deployment on mobile devices with limited resources.

This strategic trade-off in favor of resource efficiency positions SCGNet as a compelling candidate for deployment in practical scenarios, where considerations of computational cost are pivotal. Such efficiency gains can contribute significantly to the feasibility and scalability of deploying deep learning models in resource-constrained environments.

### 4.5 Ablation study

To ascertain the individual contributions of each module within the SCGNet model to its overall performance, we conducted a series of ablation studies. These studies encompass the following scenarios: (1) SCG block without the GM module (-w/o GM), (2) SCG block without the SC module (-w/o SC), (3) Instead of applying a 3-D convolutional classification layer, a traditional classification layer is used instead, and (4) Replacing the Swish activation function with the traditional ReLU activation function(-w/o Swish).

The impact of each ablation study on the model’s performance is summarized in **Table 8**.

**Table 8:** Discriminatory results of different modules for the implementation of ablation studies on test samples.

Methods	Accuracy	FLOPs	Parameters
-w/o GM	97.43%	34.43 M	1.03 M
-w/o SC	99.47%	61.98 M	1.85 M
-w/o 3-D Conv	99.52%	82.36 M	2.46 M
-w/o Swish	99.56%	35.57 M	1.14 M

Specifically, (1) -w/o GM exhibits a pronounced effect on recognition performance. This is attributed to the GM module’s role in facilitating the exchange of feature information among group convolutions. The absence of the GM module impedes individual group convolutions from effectively learning additional features from one another.

Contrastingly, (2) -w/o SC demonstrates minimal impact on recognition performance. However, it results in an increase in the number of parameters, along with metrics such as FLOPs, due to the use of fully connected.

Moreover, (3) -w/o 3-D Conv yields a tiny effect on recognition performance. However, the number of parameters and FLOPs are dramatically increased due to the large number of fully connected computations involved in the traditional classification layer.

Finally, (4)-w/o Swish, the Swish activation function has a smoothness that enhances the forward propagation optimization, and replacing ReLU with Swish brings about a lesser drop in FLOPs without any loss of accuracy. In addition, our investigation delves into the impact of varying strides on the model’s performance within the sparsely connected methodology. We systematically evaluate the effects of strides set at 2, 3, 5, 7, and 9, employing three key metrics: accuracy, FLOPs, and the number of parameters. The results, summarized in **Table 3**, elucidate the influence of each stride value on model performance.

Remarkably, when the stride is set to 3, the model demonstrates optimal recognition performance. As the stride increases, the computational load of the model diminishes. Simultaneously, however, there is a discernible and precipitous decline in the recognition accuracy of the model. This decline is particularly pronounced when the stride is set to 9, resulting in a precipitous drop akin to a cliff.

## 5 Conclusion

In this research, we introduce a specialized CNN for precise wheat grain classification. We propose “Group Mixing” to address information flow issues in group convolution, and “Sparsely Connected” methodology to reduce parameter redundancy, minimizing FLOPs and parameters. In addition, we have innovatively devised a new classification output layer predicated on 3-D convolution, supplanting the conventional maximum pooling layer and fully connected layer, replacing traditional classification layers without sacrificing accuracy. Drawing from the foregoing advancements, we have conceived an efficient Sparsely Connected Group Convolution Network, custom-tailored for the high-resolution classification of wheat grains.

Numerous rigorous experimental evaluations substantiate the prowess of our proposed SCGNet, which attains an impressive accuracy rate of 99.56%. Moreover, our approach is notably characterized by a parsimonious parameter count and reduced FLOPs, rendering it exceptionally suitable for deployment on mobile devices.

However, we acknowledge limitations in our dataset and SCGNet architecture. The dataset lacks diversity in wheat varieties, necessitating the acquisition of more varied datasets. SCGNet, tested in controlled high-resolution conditions, needs validation for low-resolution images from mobile devices.

The amalgamation of computer vision techniques with the automated non-destructive classification of individual wheat grains portends significant potential across diverse applications. In forthcoming endeavors, our research trajectory will encompass the collection of images representing a broader spectrum of wheat varieties and possibly other crop seeds. Building upon these comprehensive datasets, we endeavor to enhance the efficacy of the SCGNet architecture, with a particular focus on bolstering its robustness, reducing its parameter count and FLOPs, and venturing into deployment on mobile terminals. The latter imposes stringent constraints on model size, an exigent challenge we are poised to tackle.

## Data availability statement

The original contributions presented in the study are included in the article/supplementary materials, further inquiries can be directed to the corresponding author.

## Author contributions

XS: Writing – original draft, Writing – review & editing. YL: Writing – review & editing. GL: Writing – original draft, Writing – review & editing. SJ: Writing – review & editing. WYZ: Writing – review & editing. ZL: Writing – review & editing. WDZ: Writing – review & editing.
